# Clinical Significance of Coiled-Coil Domain-Containing Protein 25 Expression in Esophageal Squamous Cell Carcinoma

**DOI:** 10.1245/s10434-025-16964-z

**Published:** 2025-02-14

**Authors:** Takafumi Suzuki, Hironori Tsujimoto, Takanori Watanabe, Yusuke Ishibashi, Seiichiro Fujishima, Yujiro Itazaki, Risa Kariya, Naoyuki Uehata, Hanae Shinada, Satsuki Mochizuki, Yoji Kishi, Kimiya Sato, Hideki Ueno

**Affiliations:** 1 Department of Surgery, National Defense Medical College, Tokorozawa, Japan; 2 Department of Basic Pathology, National Defense Medical College, Tokorozawa, Japan

**Keywords:** Coiled-coil domain-containing protein 25, Esophageal squamous cell cancer, Neutrophil extracellular traps, Neutrophil, Postoperative infectious complication

## Abstract

**Background:**

Neutrophil extracellular traps (NETs) have been implicated in cancer progression by enhancing cancer cell motility through the coiled-coil domain-containing protein 25 (CCDC25).

**Objective:**

This study aimed to evaluate the prognostic value of CCDC25 expression in patients with esophageal squamous cell carcinoma (ESCC).

**Method:**

Tissue specimens from patients who underwent radical esophagectomy for ESCC were analyzed to investigate the relationship between CCDC25 expression, clinicopathological features, and prognosis. Western blotting was conducted to compare CCDC25 expression in tumor and non-tumorous tissues (*n* = 4). Immunohistochemical staining was performed to categorize patients into two groups based on CCDC25 expression: CCDC25^high^ (*n* = 80) and CCDC25^low^ (*n* = 40).

**Results:**

CCDC25 expression was significantly higher in tumor tissues compared with non-tumorous tissues (*p* = 0.006). Patients in the CCDC25^high^ group had a higher likelihood of poorly differentiated tumors; however, no significant differences were observed in the other clinicopathological features between the two groups. High CCDC25 expression was associated with significantly lower relapse-free survival (RFS) rate (*p* = 0.03) but not overall survival (OS) [*p* = 0.07]. Multivariate analysis revealed that high CCDC25 expression and tumor depth were independent predictors of both RFS and OS. Furthermore, high CCDC25 expression was significantly associated with worse prognosis in patients with high postoperative neutrophil counts (≥ 9600/µL) and those with postoperative infectious complications.

**Conclusions:**

High expression of CCDC25 was identified as an unfavorable prognostic factor in patients with ESCC, particularly in those with elevated postoperative neutrophil counts. Targeting CCDC25 could potentially improve prognosis in specific subgroups of patients with ESCC.

**Supplementary Information:**

The online version contains supplementary material available at 10.1245/s10434-025-16964-z.

Esophageal cancer is ranked the 11th most common cancer globally and 7th leading cause of cancer-related death, with approximately 510,000 morbidities and 445,000 associated mortalities annually.^1^ Despite advances in minimally invasive surgery, such as thoracoscopic and robotic surgery, radical surgery for esophageal cancer remains invasive, often associated with significant quality-of-life deterioration and poor prognosis.^2^ Cancer-related inflammation has been reported to contribute to tumor growth and progression in various types of cancer, and we previously showed that immunoinflammatory measures, including neutrophil-to-lymphocyte ratio, can predict long-term outcomes in esophageal cancer.^3,4^ However, the precise mechanisms by which neutrophils influence the tumor microenvironment remain unclear.

Neutrophils, a crucial component of the innate immune system, play a dual role in cancer. While these cells typically combat microbes and tumor cells,^5^ evidence suggests their potential for immunosuppressive function in tumors. As such, the impact of elevated neutrophil on the outcomes of patients with cancer remain controversial.^5,6^

A novel aspect of neutrophil function involves the neutrophil extracellular traps (NETs), composed of chromatin DNA filaments coated with granule proteins, which are formed to trap microorganisms.^7^ Recent studies have reported that the DNA component of NETs (NET-DNA) may be implicated in cancer metastasis. Notably, Yang et al. showed that the transmembrane protein coiled-coil domain-containing protein 25 (CCDC25) acts as a NET-DNA receptor on cancer cells and senses extracellular DNA, subsequently activating the ILK-β-parvin pathway to enhance cancer cell motility.^8^ Furthermore, their study demonstrated that CCDC25 expression in human umbilical vein endothelial cells promoted angiogenesis within the tumor microenvironment and growth upon NET stimulation.^9,10^

While high levels of CCDC25 expression have been linked to liver metastasis in colon cancer and poor prognosis in breast cancer,^8^ no studies have investigated its impact and prognostic implications in esophageal squamous cell carcinoma (ESCC). Therefore, this study aimed to investigate the prognostic significance of CCDC25 expression in patients with ESCC, focusing on its association with elevated postoperative neutrophil counts.

## Methods

### Study Design and Setting

This retrospective, single-center study was approved by the Internal Review Board of the National Defense Medical College, Tokorozawa, Japan (permission number 4687), and the study protocol adhered to the Declaration of Helsinki guidelines. Written informed consent was obtained from all participants prior to study enrollment.

### Patients

The study included 120 Japanese patients (102 males [85%], 18 females [15%]; average age 68.9 ± 8.4 years) with ESCC who underwent curative esophagectomy between January 2009 and December 2015. Curative esophagectomy was performed in all cases (thoracotomy: 41; thoracoscopy: 79 cases), and 57 received neoadjuvant chemotherapy (NAC) with two cycles of 5-fluorouracil and cis-diamminedichloroplatinum. Tumor staging followed the 8th edition of the Union for International Cancer Control tumor node metastasis (TNM) classification system.^11^ Postoperative complications were defined as grade ≥ 2 adverse events according to the Clavien–Dindo (CD) classification,^12^ with postoperative infectious complications specifically defined as experiencing one or more of the following adverse events: pneumonia, pyothorax, acute respiratory distress, anastomotic leakage, surgical site infection, and other surgical-related infections.

### Evaluation of Postoperative Neutrophil Count

Peripheral blood testing was performed to determine the maximum neutrophil count (neutrophil count^max^) for all participants immediately post-surgery and on alternate days for up to 7 days postoperatively. Patients were then categorized into two groups based on the median neutrophil count of 9600/µL. The trend of postoperative neutrophil counts and the timing of the highest count are presented in electronic supplementary Fig. 1.

### Western Blotting

Western blotting was performed using fresh-frozen tumor and non-tumorous tissues from four recent cases. Total proteins were extracted using radioimmunoprecipitation assay (RIPA) buffer. Protein extracts were subsequently resolved in 10% sodium dodecyl sulfate-polyacrylamide gel electrophoresis (SDS-PAGE) gel and electro-transferred to polyvinylidene difluoride (PVDF) membranes (GE Healthcare, Tokyo, Japan) in ice water. Membranes were blocked using Block-Ace (DS Pharma Biomedical, Osaka, Japan) for 2 h at room temperature and incubated overnight at 4 °C with rabbit polyclonal anti-CCDC25 (1:1000, 1209-1-AP, ProteinTech) after gentle shaking. Following phosphate-buffered saline washing using Tween-20 (PBS-T), membranes were incubated with horseradish peroxidase-conjugated anti-mouse secondary antibodies (Envision+ System-HRP; Dako) for 2 h at room temperature. Detection was performed using enhanced chemiluminescence detection reagents (SuperSignal West Dura Extended Duration Substrate; Thermo Fisher Scientific Inc, Tokyo, Japan), and images were acquired using the ImageQuant LAS 4000 system (GE Healthcare). Anti-glyceraldehyde-3-phosphate dehydrogenase (GAPDH) antibodies (1:1000 dilution; catalog no. ab125247; Abcam, Cambridge, MA, USA) were used to ensure that an equal amount of protein was loaded as a control. Furthermore, Image J software (ImageJ Launcher, Broken Symmetry Software, Bristol, UK) was used to quantify protein band intensities.

### Immunohistochemical Staining and Evaluation

CCDC25 expression was evaluated using immunohistochemical (IHC) staining, as previously described. Briefly, 3 mm central tumor sections were deparaffinized, rehydrated in a graded ethanol series, subjected to antigen retrieval with a pH 9.0 buffer (Nichirei Bioscience, Tokyo, Japan), and autoclaved at 121 for 15 min. Sections were then incubated overnight at 4 °C with rabbit monoclonal anti-CCDC25 antibody (all 1:100, 1209-1-AP, ProteinTech) and subsequently with a dextran polymer reagent combined with secondary antibody (Envision+ System-HRP; Dako). Two pathologists (TW and KS), who were blinded to clinical outcomes, evaluated staining intensity and percentage of stained cells. In discrepant cases, a consensus was reached after re-evaluation.

IHC was performed for 120 cases and scored using the human epidermal growth factor receptor 2 (HER2) IHC scoring system (American Society of Clinical Oncology/College of American Pathologists guidelines).^13^ A score of 3+ indicated circumferential and intense CCDC25 staining in > 10% of tumor cells; a score of 2+ indicated weak-to-moderate complete membrane staining in > 10% of tumor cells, or intense whole-membrane CCDC25 staining in < 10% of tumor cells; a score of 1+ indicated non-circumferential and slight staining in > 10% of tumor cells, and a score of 0 was given for no staining, or non-circumferential and slight staining in < 10% of tumor cells. Patients with IHC scores of 3+ or 2+ were placed in the CCDC25^high^ group, whereas those with IHC scores of 1+ or 0 were placed in the CCDC25^low^ group.

### Statistical Analysis

Data were expressed as means ± standard deviations. Statistical comparison was performed using the Wilcoxon and Chi-square tests, as appropriate. Prognostic implications were analyzed using Cox’s univariate and multivariate proportional hazards models, with hazard ratios (HRs) and 95% confidence intervals (CIs) reported. Survival probabilities were evaluated using the Kaplan–Meier method, and differences were determined using the log-rank test. All statistical analyses were performed using JMP 14 (SAS Institute Inc., Cary, NC, USA), and statistical significance was set at *p* < 0.05.

## Results

### Evaluation of Coiled-Coil Domain-Containing Protein 25 (CCDC25) Expression

**Fig. 1:**
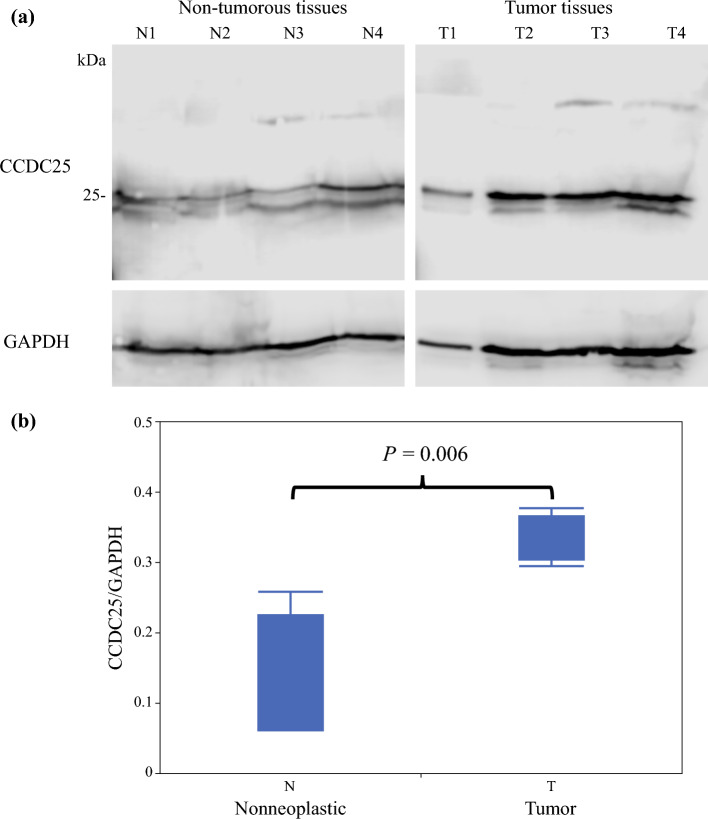
**a** Comparison of CCDC25 expression between tumor and non-tumorous tissues in ESCC using Western blotting. **b** Western blotting reveals significantly elevated CCDC25 expression in tumor tissues than in non-tumorous tissues. *CCDC25* coiled-coil domain-containing protein 25, *GAPDH* glyceraldehyde-3-phosphate dehydrogenase, *ESCC* esophageal squamous cell carcinoma

**Fig. 2:**
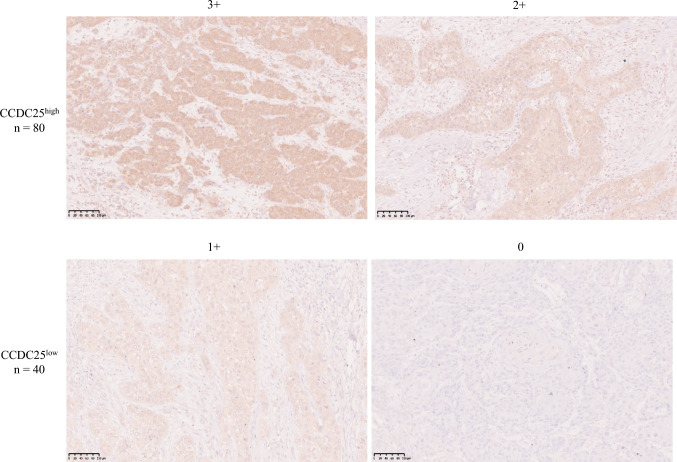
Immunohistochemical staining assessment of CCDC25. The representative images show specimens with scores of 3+, 2+, 1+, and 0 (200×). Scores of 3+ or 2+ were defined as CCDC25^high^, while scores of 1+ and 0 were defined as CCDC25^low^. *CCDC25* coiled-coil domain-containing protein 25

Western blotting analysis revealed that CCDC25 expression was significantly higher in tumor tissues compared with non-tumorous tissues (*p* = 0.006) [Fig. 1]. IHC staining of the 120 patients exhibited the following scores: 3+ in 17 patients (14.2%), 2+ in 63 patients (52.5%), 1+ in 28 patients (23.3%), and 0 in 12 patients (10.0%). Based on these scores, 80 patients were placed in the CCDC25^high^ group, while 40 were placed in the CCDC25^low^ group (Fig. 2).

### Patient Clinicopathological Features

Table 1 summarizes the clinicopathological characteristics of both groups. Poor differentiation was more frequent in the CCDC25^high^ group compared with the CCDC25^low^ group (16.3% vs. 5.0%; *p* = 0.079), although this difference was not statistically significant. No significant differences were observed between the two groups in terms of age, sex, tumor depth (pT), lymph node metastasis (pN), lymphatic invasion (Ly), vascular invasion (V), and NAC administration. Moreover, the neutrophil count^max^ of both groups was comparable (11,079 ± 5154/µL vs. 10,997 ± 3959/µL; *p* = 0.931). Additionally, no significant differences were noted in surgical factors, including surgical procedure, lymph node dissection of the cervical area, operating time, bleeding, and frequency of postoperative infectious complications.

**Table 1:** Comparison of clinicopathological features depending on the expression of CCDC25

		Total		CCDC25^high^		CCDC25^low^		*p*-Value
		[*n* = 120]	(%)	[*n* = 80]	(%)	[*n* = 40]	(%)	
*Clinicopathological* *features*								
Age, years (mean ± SD)		68.9 ± 8.4		69.6 ± 7.0		67.5 ± 10.6		0.202
Sex								
Male		102	(85.0)	69	(86.3)	33	(82.5)	0.588
Female		18	(15.0)	11	(13.8)	7	(17.5)	
Alcohol								
Yes		87	(72.5)	56	(70.0)	31	(77.5)	0.386
No		33	(27.5)	24	(30.0)	9	(22.5)	
Brinkman Index		524 ± 621		530 ± 655		513 ± 559		0.884
Tumor location								
Ut		17	(14.2)	11	(13.8)	6	(15.0)	0.653
Mt		52	(43.3)	37	(46.3)	15	(37.5)	
Lt Ae		51	(42.5)	32	(40.0)	19	(47.5)	
Depth of tumor								
T1/2		51	(42.5)	32	(40.0)	19	(47.5)	0.433
T3/4		69	(57.5)	48	(60.0)	21	(52.5)	
Lymph node metastasis			0.0					
No		47	(39.2)	28	(35.0)	19	(47.5)	0.186
Yes		73	(60.8)	52	(65.0)	21	(52.5)	
Lymphatic invasion								
0		36	(30.0)	21	(26.3)	15	(37.5)	0.205
1		84	(70.0)	59	(73.8)	25	(62.5)	
Vascular invasion								
0		24	(20.0)	17	(21.3)	7	(17.5)	0.628
1		96	(80.0)	63	(78.8)	33	(82.5)	
Degree of differentiation								
Not poorly		105	(87.5)	67	(83.8)	38	(95.0)	0.079
Poorly		15	(12.5)	13	(16.3)	2	(5.0)	
Neoadjuvant therapy								
NAC		57	(47.5)	37	(46.3)	20	(50.0)	0.698
None		63	(52.5)	43	(53.8)	20	(50.0)	
Maximal postoperative CRP (mg/dL)								
		16.9 ± 7.3		17.4 ± 7.0		15.9 ± 7.7		0.299
Maximal postoperative neutrophil counts (/µL)		11,052 ± 4772		11,079 ± 5154		10,997 ± 3959		0.931
*Surgical* *factors*								
Operation procedure								
Thoracotomy		41	(34.2)	28	(35.0)	13	(32.5)	0.786
Thoracoscopy		79	(65.8)	52	(65.0)	27	(67.5)	
Lymph node dissection of the cervical area								
Yes		46	(38.3)	33	(41.3)	13	(32.5)	0.353
No		74	(61.7)	47	(58.8)	27	(67.5)	
Operation time, min		472 ± 100		469 ± 83		480 ± 129		0.576
Bleeding, mL		510 ± 662		447 ± 505		637 ± 894		0.140
Infectious complications		57	(47.5)	40	(50.0)	17	(42.5)	0.438
Pulmonary complications		24	(20.0)	18	(22.5)	6	(15.0)	0.488
Anastomotic leakage		15	(22.5)	17	(21.3)	10	(25.0)	
Others		1	(1.7)	1	(1.3)	1	(2.5)	

*CCDC25* coiled-coil domain-containing protein 25, *SD* standard deviation, *Ut* upper thoracic esophagus, *Mt* middle thoracic esophagus, *Lt* lower thoracic esophagus, *Ae* abdominal esophagus, *CRP* C-reactive protein, *NAC* neoadjuvant chemotherapy

### Univariate and Multivariate Analyses for Recurrence-Free and Overall Survival

Univariate analysis demonstrated that pT, pN, Ly, V, NAC, and CCDC25^high^ were significantly correlated with relapse-free survival (RFS). Furthermore, multivariate analysis identified pT (HR 3.85, 95% CI 2.04–7.58; *p* < 0.001) and CCDC25^high^ (HR 1.78, 95% CI 1.08–3.04, *p* = 0.023) [Table 2]. For overall survival (OS), multivariate analysis revealed that pT (HR 3.45, 95% CI 1.78–7.13; *p* < 0.001) and CCDC25^high^ (HR 1.72, 95% CI 1.01–3.07; *p* = 0.047) were independent prognostic factors. Conversely, neutrophil count^max^ (≥ 9600/µL) and postoperative infectious complications (CD grade ≥ 2) were not identified as independent prognostic factors for either RFS or OS (Table 3).

**Table 2:** Uni- and multivariate analyses for recurrence-free survival

Variable	Univariate		Multivariate	
	HR (95% CI)	*p-*Value	HR (95% CI)	*p-*Value
Depth of tumor				
1.2	Ref.	< 0.001	Ref.	< 0.001
3.4	3.97 (2.40–6.86)		3.85 (2.04–7.58)	
Lymph node metastasis				
No	Ref.	0.002	Ref.	0.273
Yes	2.15 (1.33–3.59)		1.47 (0.75–3.13)	
Lymphatic invasion				
No	Ref.	0.015	Ref.	0.453
Yes	1.89 (1.12–3.35)		1.36 (0.61–3.07)	
Vascular invasion				
No	Ref.	0.012	Ref.	0.731
Yes	2.12 (1.16–4.26)		1.14 (0.55–2.51)	
Degree of differentiation				
Not poorly	Ref.	0.054		
Poor	1.93 (0.99–3.46)			
Neoadjuvant chemotherapy				
No	Ref.	0.003	Ref.	0.960
Yes	1.99 (1.26–3.21)		1.01 (0.593–1.691)	
CCDC25				
1+/0	Ref.	0.027	Ref.	0.023
3+/2+	1.73 (1.06–2.91)		1.78 (1.08–3.04)	
Maximal postoperative neutrophil count				
9600 >	Ref.	0.127		
≥ 9600	1.42 (0.91–2.24)			
Infectious complication				
No	Ref.	0.145		
Yes	1.40 (0.89–2.21)			

*HR* hazard ratio, *CI* confidence interval, *Ref* reference, *CCDC25* coiled-coil domain-containing protein 25

**Table 3:** Uni- and multivariate analyses for overall survival

Variable	Univariate		Multivariate	
	HR (95% CI)	*p-*Value	HR (95% CI)	*p-*Value
Depth of tumor				
1.2	Ref.	< 0.001	Ref.	< 0.001
3.4	3.71 (2.18–6.65)		3.45 (1.78–7.13)	
Lymph node metastasis				
No	Ref.	0.004	Ref.	0.390
Yes	2.10 (1.26–3.63)		1.42 (0.65–3.44)	
Lymphatic invasion				
No	Ref.	0.018	Ref.	0.579
Yes	1.92 (1.11–3.55)		0.77 (0.53–2.93)	
Vascular invasion				
No	Ref.	0.028	Ref.	0.664
Yes	2.06 (1.07–4.47)		1.20 (0.53–2.93)	
Degree of differentiation				
Not poorly	Ref.	0.090		
Poor	1.88 (0.90–3.55)			
Neoadjuvant chemotherapy				
No	Ref.	0.029	Ref.	0.845
Yes	1.88 (0.90–3.55)		0.95 (0.55–1.65)	
CCDC25				
1+/0	Ref.	0.066	Ref.	0.047
3+/2+	1.62 (0.97–2.83)		1.72 (1.01–3.07)	
Maximal postoperative neutrophil count				
9600 >	Ref.	0.032	Ref.	0.061
≥ 9600	1.70 (1.04–2.79)		1.63 (0.98–2.78)	
Infectious complications				
No	Ref.	0.011	Ref.	0.085
Yes	1.87 (1.16–3.07)		1.57 (0.94–2.66)	

*HR* hazard ratio, *CI* confidence interval, *Ref*. reference, *CCDC25* coiled-coil domain-containing protein 25

### Impact of Postoperative Neutrophil Counts and Infectious Complications on the Prognostic Value of CCDC25

**Fig. 3:**
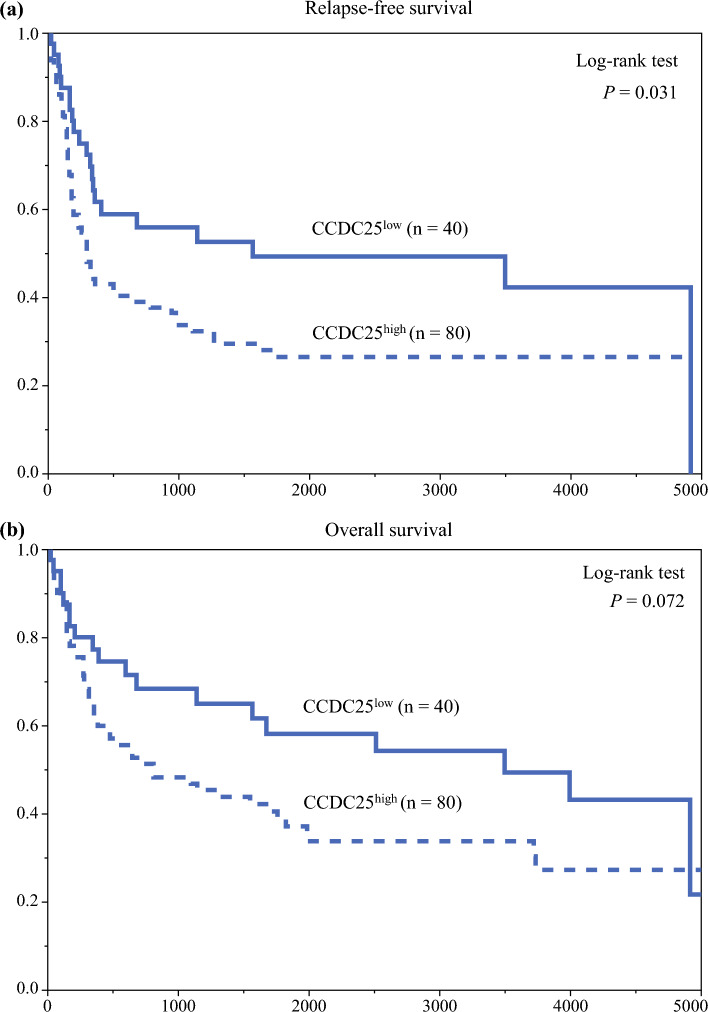
**a** Relapse-free survival and **b** overall survival according to CCDC25 expression. *CCDC25* coiled-coil domain-containing protein 25

**Fig. 4:**
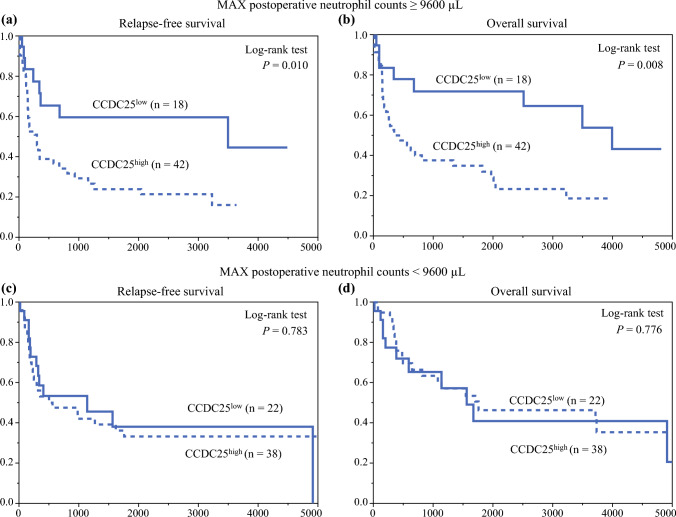
Subgroup analysis in the group with a high neutrophil count^max^: **a** relapse-free survival and **b** overall survival according to CCDC25 expression. Subgroup analysis in the group with a low neutrophil count^max^: **c** relapse-free survival and **d** overall survival according to CCDC25 expression. *CCDC25* coiled-coil domain-containing protein 25

**Fig. 5:**
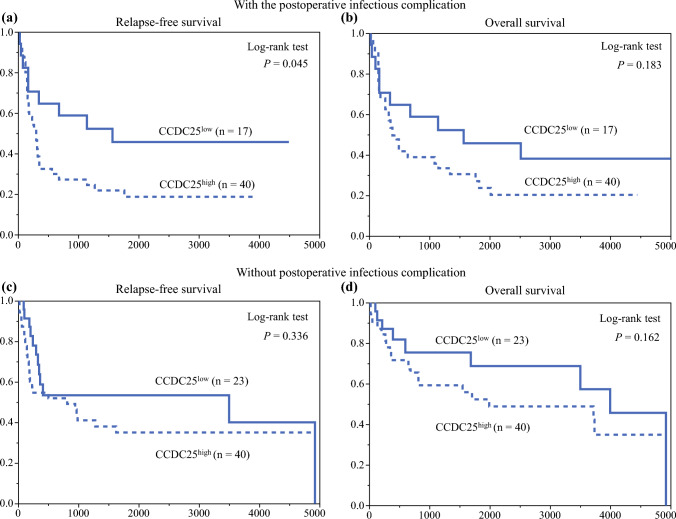
Subgroup analysis in the group with postoperative infectious complications: **a** relapse-free survival and **b** overall survival according to CCDC25 expression. Subgroup analysis in the group without postoperative infectious complications: **c** relapse-free survival and **d** overall survival according to CCDC25 expression. *CCDC25* coiled-coil domain-containing protein 25

Figure 3 illustrates the stratification of RFS by CCDC25 expression. The CCDC25^high^ group exhibited significantly worse RFS than the CCDC25^low^ group (*p* = 0.031), with 5-year RFS rates of 26.5% and 49.3%, respectively; however, no significant difference in OS was reported between the two groups (*p* = 0.072) [Fig. 3b], with 5-year OS rates of 37.1% and 58.1%, respectively. Subgroup analysis of postoperative neutrophil counts revealed that in patients with neutrophil count^max^ ≥ 9600/µL, CCDC25^high^ was associated with significantly worse RFS (*p* = 0.034) and OS (*p* = 0.028) [Figs. 4a, b], whereas CCDC25 expression did not significantly impact RFS or OS in patients with neutrophil count^max^ < 9600/µL (Figs. 4c, d). Subgroup analysis of postoperative infectious complications showed that CCDC25^high^ was associated with significantly worse RFS (*p* = 0.045) but not OS (*p* = 0.183) [Figs. 5a, b] among patients with such complications. In contrast, CCDC25 expression was not associated with RFS or OS in patients without postoperative infectious complications (Figs. 5c, d).

## Discussion

This study investigated the role of CCDC25 expression in ESCC and its relationship with prognosis following radical esophagectomy. Our findings suggest that high CCDC25 expression in ESCC was associated with poor RFS, serving as an independent prognostic factor. This was significantly observed in patients with postoperative neutrophil count^max^ ≥ 9600/μL and those with postoperative infectious complications compared with patients without these characteristics, indicating the role of CCDC25 in promoting cancer progression.

Previous studies have demonstrated that patients with high postoperative inflammatory states, such as increased interleukin (IL)-6 and IL-8 levels, have poor prognosis in esophageal cancer.^14–16^ Nishi et al. reported that high CXCR2 expression, an IL-8 receptor, correlated with poor prognosis, especially in patients with postoperative infectious complications.^17^ These infections contribute to elevated serum IL-8 levels during the first postoperative week, potentially triggering mechanisms that promote cancer progression.^18^ Our findings support this notion, as high CCDC25 expression was associated with poor outcomes only in the presence of elevated postoperative neutrophil counts and postoperative infectious complications. Similarly, Bostock et al. demonstrated that prolonged neutrophilia was associated with worse outcomes following esophagectomy.^18,19^ These results may explain the link between high neutrophil levels and worse prognosis after operative treatment for esophageal cancer. In fact, our previous study showed that postoperative inflammation and infection were associated with poor cancer prognosis, identifying postoperative infectious complications as independent predictors of OS.^3,4,20,21^ While not as significant as infectious complications, surgical trauma itself can also increase cytokine production and promote tumor proliferation of cancer cells.

NETs are released from activated neutrophils following stimulation from signaling molecules, including lipopolysaccharide and IL-8.^9^ Acting as a key factor in NET formation, IL-8 induces neutrophil migration in the tumor microenvironment. Surgical stress triggers the release of inflammatory cytokines, such as serum IL-8,^22^ resulting in increased NET formation during the postoperative period. Additionally, Xia et al.^23^ reported that abdominal infectious complications after gastrectomy induced NET formation, and NETs trapped free gastric cancer cells to form NET-gastric cancer clusters in which NETs facilitated gastric cancer cell metastasis. In our study, high CCDC25 expression was correlated with poor RFS only in the presence of postoperative infectious complications, suggesting that the prognostic impact of postoperative inflammation and complications in patients with cancer depend on the levels of CCDC25 expression. Our results are consistent with the research results reported by Xia et al. We speculated that CCDC25 highly expressed tumor cells in the peripheral blood after curative resection are susceptible to the effects of cancer metastasis promotion by NETs induced by surgical stress and postoperative complications. Although NETs were not directly measured in this study, our findings indicate that patients with CCDC25-positive esophageal cancer may be more susceptible to metastasis promotion by NETs.

CCDC25 is a transmembrane protein expressed in hepatocytes and muscle cells with low tissue specificity.^24^ This study showed that compared with normal esophageal tissue, ESCC tissue exhibited higher CCDC25 expression. Based on the Human Protein Atlas, CCDC25 expression levels vary across different cancers, with negative to minimal expression in head and neck cancers, < 10% in renal cancer, approximately 20% in gastric cancer, and > 60% in breast and colon cancers; however, the frequency of CCDC25 expression in esophageal cancer was not reported.^24^ The role of CCDC25 in the promotion of cancer metastasis may be different across cancer types.^25^ The present study showed that CCDC25 expression did not stratify prognosis in patients with low postoperative neutrophil counts, which suggests that with fewer NETs released in this group, CCDC25 may have a limited impact on prognosis.

Despite the insights provided by this study, several limitations must be acknowledged. First, this was a retrospective, single-center study that included a relatively small sample size. Second, NETs were not directly measured in this study, necessitating further investigation into the relationship between neutrophil activation, NET formation, and prognosis in CCDC25-positive esophageal cancer. Lastly, the method of CCDC25 evaluation may not have been optimal, since CCDC25 expression was subjectively evaluated by pathologists using the HER2 IHC scoring system.

## Conclusion

High CCDC25 expression in ESCC was associated with poor RFS and was an independent prognostic factor for RFS. Although CCDC25 expression did not affect prognosis in cases with relatively low postoperative neutrophil counts and without postoperative infectious complications, it was associated with poor prognosis in patients with high neutrophil activation. These findings indicate a potential relationship between neutrophil activation, NET formation, and prognosis in CCDC25-positive esophageal cancer, warranting further investigation.

## Supplementary Information

Below is the link to the electronic supplementary material.

Supplementary file1 (TIF 489 KB)
