# Supplementary figures and images for: Clinical Significance of Coiled-Coil Domain-Containing Protein 25 Expression in Esophageal Squamous Cell Carcinoma
Source: Ann Surg Oncol. 2025 Feb 14;32(5):3839–50. doi: 10.1245/s10434-025-16964-z (PMC11976332; doi:10.1245/s10434-025-16964-z)

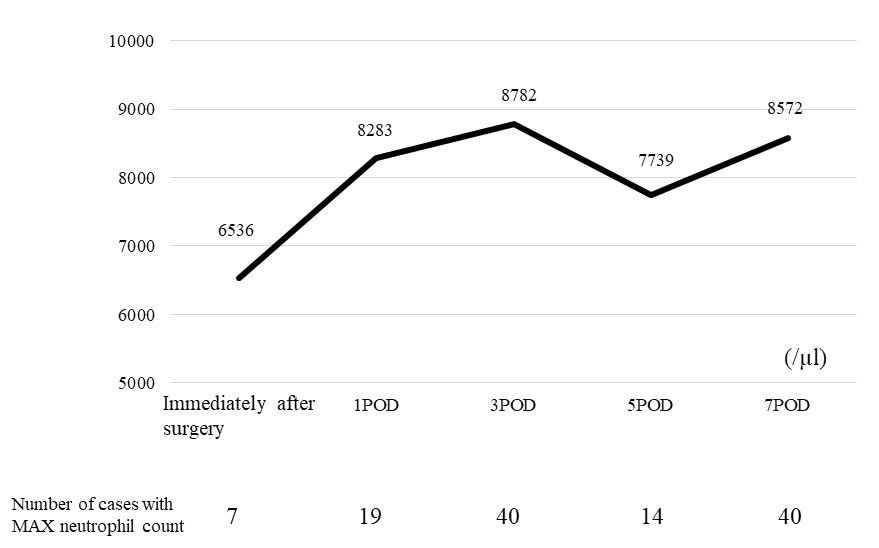

Supplement: Supplementary file 1 — Supplementary file1 (TIF 489 KB) [file 10434_2025_16964_MOESM1_ESM.tif]
